# Maltreatment Related Trauma Symptoms Affect Academic Achievement through Cognitive Functioning: A Preliminary Examination in Japan

**DOI:** 10.3390/jintelligence5040032

**Published:** 2017-09-21

**Authors:** Kohske Ogata

**Affiliations:** Osaka Prefectural Government Kishiwada Child-Family Center, Kishiwada 596-0043, Japan; koh-ske@sakai.zaq.ne.jp; Tel.: +81-90-1130-1484

**Keywords:** trauma symptoms, academic achievement, cognitive functioning, covariance structure analysis, maltreated children

## Abstract

Child abuse and neglect could have some deleterious impacts on both intellectual and academic performance of school students. The aim of this study was to examine relationships among child maltreatment, trauma symptoms, cognitive functioning, and academic achievement. Data were collected from child guidance centers, where maltreated children were substantiated, assessed, evaluated, protected, and treated clinically. The selection criteria for subjects included Japanese children (1) who had a history of maltreatment; (2) whose IQs were measured using the Kaufman Assessment Battery for Children second edition (KABC-II); and (3) whose traumatic stress was evaluated using the Trauma Symptom Checklist for Children alternate version (TSCC-A). Covariance structure analysis showed the model that explains the relations of trauma symptom (measured by TSCC-A) on academic achievement (measured by KABC-II) as being intervened by cognitive functioning (measured by KABC-II).

## 1. Introduction

Child maltreatment can have an adverse impact on a child’s development. Veltman and Browne’s [1] review of studies published over the past 30 years revealed delayed language and/or cognitive development, low intelligence quotient (IQ), and poor school performance in maltreated children. Glaser [2] also reviewed neurobiological studies and indicated that child abuse and neglect have a negative impact on brain development. Developmental problems could exacerbate maladjustment in school settings for children who experience abuse and neglect. There are strong relationships between psychometric intelligence and school achievement. Through an English longitudinal study examining over 70,000 children, Deary, Strand, Smith, and Fernandes [3] revealed that the correlation between IQ and educational achievement is around 0.81.

As the relationship between IQ and achievement might suggest, child abuse and neglect have deleterious effects on not only intellectual development based on brain maturity but also academic achievement and school performance. Eckenrode, Laird, and Doris [4] compared 420 maltreated children with 420 matched non-maltreated counterparts and found that maltreated children had lower scores on standardized tests than the matched comparisons. In further analyses of Kendall-Tackett and Eckenrode [5] using the same data set, neglected children performed more poorly than their matched students in terms of academic achievement. Comparing across physically abused (*n* = 22), neglected (*n* = 47), and non-maltreated children (*n* = 70), Kurtz, Gaudin, Wodarski, and Howing [6] indicated that the neglected group displayed more severe academic delays than the control group. These findings suggest that poor academic achievement is predicted by impaired intellectual development due to maltreatment. Although the ways in which intelligence predicts educational achievement seem clear [3], the mechanism by which child maltreatment affects intellectual development remains unclear.

One possible factor that might explain the path from maltreatment to delayed childhood development may be trauma-related symptoms. In an investigation of 299 children aged 6 to 7 years old, Delaney-Black et al. [7] examined the relationship between violence exposure and trauma-related symptoms, and also measured IQ using the Wechsler Primary and Preschool Scale of Intelligence–Revised. They obtained a significant negative effect of violence exposure on IQ in a multiple regression model, but did not detect such an effect for trauma-related distress. Saltzman, Weems, and Carrion [8] assessed 59 children aged 7 to 14 years old using the Wechsler Abbreviated Scales of Intelligence (WASI), and indicated that posttraumatic stress disorder (PTSD) symptomatology could predict full-scale and verbal IQs but not performance IQ. DePrince, Weinzierl, and Combs [9] examined the executive functioning of 110 children after exposure to traumatic events. Comparisons showed that the familial trauma group was lower than non-familial trauma and no trauma groups on IQ, working memory, and processing speed. Nooner and Leaberry [10] studied 20 children using the Delis–Kaplan Executive Functions System and the Trauma Symptom Checklist for Children (TSCC), and found that both depression and anxiety mediated the relationships between trauma symptoms and executive functioning. This pilot finding suggests that improvement of depression and anxiety symptoms in children post-trauma could strengthen children’s executive functioning. These findings show that trauma-related symptoms could indeed be associated with cognitive functioning.

The aim of this study was to examine relationships among child maltreatment, trauma symptoms, cognitive functioning, and academic achievement. The present study adopted the Cattel–Horn–Carrol (CHC) theory to measure both cognitive functioning and academic achievement. The CHC theory were based on the enormous factor analytic researches and provided a framework of broad ability domains: fluid reasoning (G*f*), crystalized intelligence (G*c*), short-term memory (G*sm*), visual processing (G*v*), auditory processing (G*a*), long-term storage and retrieval (G*lr*), processing speed (G*s*), reaction and decision speed (G*t*), reading and writing (G*rw*), quantitative knowledge (G*q*), and so forth [11]. Although there have been some conflicting findings, I sought to construct a path diagram that represents a direction from trauma symptoms to academic achievement mediated by cognitive functioning. In short, my hypothesis for testing using covariance structure analysis was that trauma due to maltreatment leads to impaired cognition, which in turn leads to reduced achievement.

## 2. Materials and Methods

I collected data from child guidance centers on Japanese children (1) who had a history of maltreatment; (2) whose IQs were measured using the Kaufman Assessment Battery for Children second edition (KABC-II) [12]; and (3) whose traumatic stress was evaluated using the TSCC alternate version (TSCC-A) [13], which excluded sexual symptom items from the original TSCC, because the sexual symptom items were not normed in the Japanese standardization study. Child guidance centers in Japan represent a public agency where maltreated children are substantiated, assessed, evaluated, protected, and treated clinically by both caseworkers and child psychologists.

The KABC-II (Japanese version) was standardized in 2013 and differs from the original version in that achievement scales are included [14,15]. In the Japanese standardization study, the achievement scales included in the previous version (K-ABC) were retained in the KABC-II, because there are relatively few achievement tests which have been scientifically standardized for children in Japan. The KABC-II Japanese version thus measures G*lr*, G*sm*, G*v*, G*f*, G*c*, G*q*, G*rw*, and general intelligence (g) according to the CHC theory. The former four scales (G*lr*, G*sm*, G*v*, and G*f*) comprise the Mental Processing Index, representing cognitive functioning, and the latter three scales (G*c*, G*q*, and G*rw*) comprise achievement, representing academic performance.

The TSCC-A Japanese version was standardized in 2009 and includes 44 items that comprise two validity scales (under-response and hyper-response) and five symptom scales: anxiety (ANX), depression (DEP), anger (ANG), posttraumatic stress (PTS), and dissociation (DIS).

I obtained final data from 55 children (29 girls and 26 boys) aged from 8 to 15 (*M* = 12, *SD* = 2) and confirmed that the TSCC-A validity scale scores (under-response and hyper-response) were within average levels. The forms of child maltreatment substantiated in the present study included physical abuse, sexual abuse, psychological abuse, and neglect. The sampled children all had a history of one or more forms of maltreatment. These data were collected as part of routine clinical practice, and informed consent was obtained from parents, caregivers, and/or the children themselves. Both the KABC-II and TSCC-A were administered by child psychologists who had worked for more than 10 years in child guidance centers.

In the present analysis, correlation coefficients were first calculated to assess relationships between cognitive functioning, achievement, and trauma symptoms. Path analysis using the observed variables was followed by covariance structure analysis with latent factors. The cognitive functioning factor was indicated via G*f*, G*v*, G*sm*, and G*lr* from the KABC-II. The achievement factor was indicated using G*c*, G*q*, and G*rw* from the KABC-II. The trauma symptoms factor was indicated via ANX, DEP, ANG, PTS, and DIS from the TSCC-A. Maximum likelihood estimation was used for both path analysis and covariance structure analysis.

## 3. Results

Descriptive statistics and bivariate analyses are shown in Table 1.

The average IQ (general intelligence) was 83.6 (*SD* = 14.6, 95% *CI* = 79.7, 87.4). All broad abilities except for G*lr* were lower than the norm, as shown using 95% *CIs*. TSCC-A symptom scores were within average levels (T < 60) and did not reach sub-clinical range (59 < T < 65), as shown in 95% *CIs*. Proportions scores falling into average ranges were as follows: 65% for ANX, 69% for DEP, 76% for ANG, 65% for PTS, and 78% for DIS. Only two zero-order negative correlations were detected between KABC-II and TSCC-A scores: G*sm* was related to both ANG and DIS.

Exploratory path analysis was performed, using the IBM SPSS Amos 22J, to identify inter- and intra-relationships between constructs, because both KABC-II and TSCC-A featured strong correlations within each scale. In the modeling procedure, insignificant paths were removed stepwise based on their *p*-values (*p* > 0.05) from the full path model where all trauma symptoms were related to all aspects of cognitive functioning, which were associated with all aspects of academic achievement (χ^2^(15) = 12.9, *p* = 0.61, CFI = 1.00, RMSEA = 0.00). However, suppression effects may have occurred due to highly correlated predictors in the path analysis using observed variables (Appendix A). Thus, I estimated a covariance structure model to investigate some predicted relationships.

**Figure 1:**
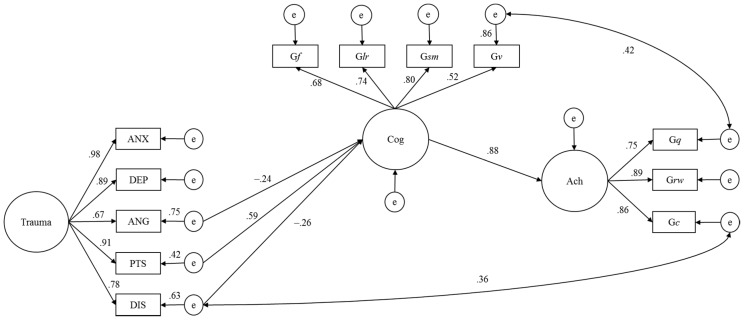
Final covariance structure model representing the relationships from trauma symptoms to academic achievement intervened by cognitive functioning: All paths are significant (*p* < 0.05). Trauma: trauma symptoms; Cog: cognitive functioning; Ach: achievement; G*lr*: long-term storage and retrieval; G*sm*: short-term memory; G*v*: visual processing; G*f*: fluid reasoning; G*c*: crystalized ability; G*q*: quantitative knowledge; G*rw*: reading and writing; ANX: anxiety; DEP: depression; ANG: anger; PTS: posttraumatic stress; DIS: dissociation.

First, the hypothetical model with trauma factors having an influence on cognitive factors was examined but the paths were insignificant. Next, relationships between the residuals of each trauma symptom and the cognitive factors were explored. The *p*-values were used to decide whether the paths from each residual were added or removed (e.g., ANG, PTS, and DIS). Modification indices were used to explore how the model fit could be improved (e.g., G*v* to G*q* and DIS to G*c*). The final covariance structure model is shown in Figure 1.

All paths were significant (*p* < 0.05) after the insignificant paths were removed stepwise. Goodness of fit indices were adequate: χ^2^(48) = 53.0, *p* = 0.29, CFI = 0.989, RMSEA = 0.044. Mediation analysis revealed that the residuals for ANG (β = −0.03, *p* = 0.78), PTS (β = 0.07, *p* = 0.52), and DIS (β = −0.15, *p* = 0.16) did not show significant direct paths to achievement factors. Therefore, in the strict sense, “mediated” could not be used to describe the final covariance structure model in Figure 1.

## 4. Discussion

The present results revealed that some trauma symptoms may directly influence cognitive functioning, and thus have indirect effects on academic achievement. Carroll and Iles [16] compared 16 undergraduate students with dyslexia with 16 controls, and found that anxiety level, as measured using the State-Trait Anxiety Inventory, was correlated with reading performance, measured using the Test of Word Reading Efficiency (*r* = −0.62, *p* < 0.01). Their finding is not consistent with the present results (see Figure 1). Djapo, Kolenovic-Djapo, Djokic, and Fako [17] investigated relationships between personality and intelligence using a sample of 105 high school students. They did not obtain a significant correlation between anxiety, measured using the 16 Personality Factor Questionnaire, and crystallized intelligence, measured by the Mill Hill Vocabulary Scale. The present covariance structure analysis shows that trauma-related anxiety may not have direct or indirect effects on cognitive abilities and achievement. Small sample size in the current study could not detect any relations between anxiety and ability-related variables.

Depression symptoms did not have indirect effects on academic performance in Figure 1. However, some earlier work has found relationships between depression and achievement [18,19]. Future studies using larger sample sizes are needed to explain the differences between prior findings and the present study. Few studies have examined the relationship between anger and achievement. In a Chinese longitudinal investigation, Zhou, Main, and Wang [20] assessed 425 children and found that anger/frustration had a negative effect on academic achievement, with this relationship mediated by externalizing problems. Although effect sizes (path coefficients) were small, the present covariance structure analysis results showed that anger had an effect on cognitive functioning. The final covariance structure model suggests that trauma-related anger may be associated with academic achievement as a function of cognitive ability, although mediation analysis showed that the indirect effect from anger to achievement was not significant. Further studies should seek to clarify in detail the effect of anger on school performance, as potentially mediated by memory related abilities (see Appendix A).

Only PTS scores showed a positive correlation with achievement scores. Figure 1 showed the indirect positive effect from PTS to Ach intervened by Cog. This finding appeared to be inconsistent with prior studies. Saltzman et al. [8] showed that PTSD deteriorated not performance IQ, representing G*f* and G*v*, but verbal IQ, representing G*c*. Furthermore, almost all previous studies have found that PTSD symptoms predict deteriorated academic performance [21,22,23]. A future study with larger sample size needs to replicate the present finding with respect to the positive effect of PTSD. Dissociation symptoms did not have significant indirect effect on achievement in the Figure 1. DePrince et al. [9] however, examined relationships among executive functioning, intelligence, and traumatic experience and obtained significant regression coefficients between dissociation symptoms and executive functioning composite scores (β = −0.28) as well as IQ scores (β = −0.23). Dissociation relates to memory impairment, particularly in trauma victims. The current examination also detected a negative path coefficient from dissociation to cognitive abilities. Abused children with dissociative symptoms might evidence difficulties with attention and concentration, or have a dysfunction in working memory [24]. In recent years, however, neuropsychological evidence using functional magnetic resonance imaging shows that trait dissociation is associated with increased working memory function [25]. Thus, further examination is needed to clarify the relationship between dissociation and memory functioning.

Clinical implications are as follows. Difficulties with emotion regulation and dissociation in maltreated children may have a detrimental influence on their cognitive abilities, particularly in memory abilities, which would also be expected to impair school performance. This finding is partially inconsistent with the current observations using the KABC-II. In general, trauma symptoms except PTS scores from the TSCC-A appear to have negative effects on cognitive functioning, with a resulting effect on academic achievement. Therefore, the present findings suggest that trauma symptoms should be routinely assessed in maltreated children when they are candidates for learning support in school settings.

There are some limitations of the present study. First, the small sample size is a major weakness and could render the present findings unstable. Second, there was no control group in this study, and thus it is unclear whether the influence of trauma on achievement is limited to maltreated victims or is relevant to the general population of children. Although this study was a preliminary pilot investigation, further studies of the relationships between child abuse and neglect, trauma responses, and cognitive/academic abilities are essential if child victims are to receive better clinical support.

## Figures and Tables

**Table 1:** Descriptive statistics and correlation coefficients for KABC-II and TSCC-A.

	1	2	3	4	5	6	7	8	9	10	11	*M*	*SD*	Skew	Kurtosis
KABC-II															
(1) G*lr*												97.8	17.8	0.24	−0.47
(2) G*sm*	0.65 **											87.4	13.8	0.38	0.07
(3) G*v*	0.36 **	0.51 **										90.1	15.1	0.47	0.19
(4) G*f*	0.45 **	0.48 **	0.33 *									85.6	14.4	1.61	3.85
(5) G*c*	0.61 **	0.56 **	0.29 *	0.54 **								86.1	14.1	1.24	2.38
(6) G*q*	0.53 **	0.62 **	0.57 **	0.44 **	0.53 **							85.1	16.8	1.03	0.15
(7) G*rw*	0.48 **	0.57 **	0.36 **	0.65 **	0.73 **	0.68 **						81.7	15.8	2.07	5.71
TSCC-A															
(8) ANX	−0.01	−0.21	−0.13	−0.16	0.04	−0.11	−0.07					55.7	13.4	0.65	−0.43
(9) DEP	−0.13	−0.24	−0.16	−0.18	−0.02	−0.11	−0.04	0.87 **				54.4	13.0	0.87	0.12
(10) ANG	−0.25	−0.34 *	−0.23	−0.20	−0.14	−0.18	−0.23	0.66 **	0.67 **			52.5	9.9	0.54	−0.12
(11) PTS	0.19	−0.03	−0.05	0.05	0.23	0.01	0.11	0.89 **	0.77 **	0.58 **		53.2	13.1	0.51	−0.11
(12) DIS	0.02	−0.27 *	−0.25	−0.16	0.07	−0.23	−0.14	0.78 **	0.68 **	0.57 **	0.78 **	52.3	13.6	1.55	2.38

* *p* < 0.05, ** *p* < 0.01; G*lr*: long-term storage and retrieval, G*sm*: short-term memory, G*v*: visual processing, G*f*: fluid reasoning, G*c*: crystrallized ability, G*q*: quantitave knowledge, G*rw*: reading and writing, ANX: anxiety, DEP: depression, ANG: anger, PTS: posttraumatic stress, DIS: dissociation.

**Figure A1:**
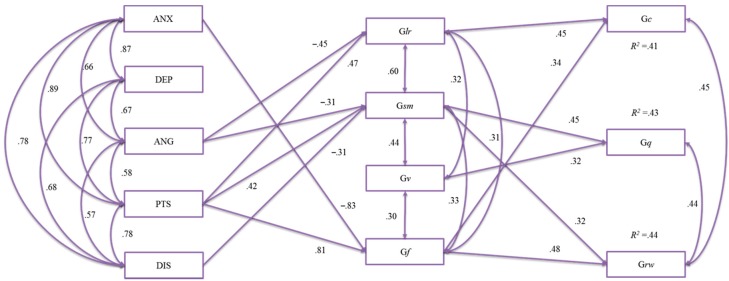
Exploratory path diagram from trauma symptoms to academic achievement intervened by cognitive functioning: All paths are significant (*p* < 0.05). Goodness of fit indices were as follows: χ^2^(35) = 38.7, *p* = 0.31, CFI = 0.992, RMSEA = 0.044.
